# The effect of a hand hygiene intervention on infections in residents of nursing homes: a cluster randomized controlled trial

**DOI:** 10.1186/s13756-021-00946-3

**Published:** 2021-05-20

**Authors:** G. R. Teesing, J. H. Richardus, D. Nieboer, M. Petrignani, V. Erasmus, A. Verduijn-Leenman, J. M. G. A. Schols, M. P. G. Koopmans, M. C. Vos, H. A. C. M. Voeten

**Affiliations:** 1 Department of Public Health, Erasmus MC, University Medical Center Rotterdam, Wytemaweg 80, 3015 CN Rotterdam, The Netherlands; 2 The Municipal Public Health Service Rotterdam-Rijnmond, Schiedamsedijk 95, 3011 EN Rotterdam, The Netherlands; 3 Municipal Public Health Service Amsterdam, Nieuwe Achtergracht 100, 1018 WT Amsterdam, The Netherlands; 4 Pieter Van Foreest, Postbus 118, 2600 AC Delft, The Netherlands; 5 Department Health Services Research, CAPHRI, Maastricht University, Universiteitssingel 40, 6229 ER Maastricht, The Netherlands; 6 Department of Viroscience, Erasmus MC, University Medical Center Rotterdam, Wytemaweg 80, 3015 CN Rotterdam, The Netherlands; 7 Department of Medical Microbiology and Infectious Diseases, Erasmus MC, University Medical Center Rotterdam, Wytemaweg 80, 3015 CN Rotterdam, The Netherlands

**Keywords:** Healthcare associated infections, Nursing homes, Hand hygiene

## Abstract

**Background:**

The primary goal of hand hygiene is to reduce infectious disease rates. We examined if a nursing home’s participation in a hand hygiene intervention resulted in residents having fewer healthcare associated infections (HAIs) when compared to nursing homes without the hand hygiene intervention.

**Methods:**

This study is a part of a cluster randomized controlled trial (RCT) in 33 nursing homes to improve hand hygiene (HANDSOME). The incidence of five illnesses was followed over 13 months: gastroenteritis, influenza-like illness, pneumonia, urinary tract infections and infections from methicillin-resistant *Staphylococcus aureus* (MRSA). Incidence rates per study arm were reported for baseline (October–December 2016) and two follow-up periods (January–April 2017, May–October 2017). HAI rates were compared in a Poisson multilevel analysis, correcting for baseline differences (the baseline infection incidence and the size of the nursing home), clustering of observations within nursing homes, and period in the study.

**Results:**

There was statistically significantly more gastroenteritis (*p* < 0.001) and statistically significantly less influenza-like illness (*p* < 0.01) in the intervention arm when compared to the control arm. There were no statistically significant differences or pneumonia, urinary tract infections, and MRSA infections in the intervention arm when compared to the control arm. In a sensitivity analysis, gastroenteritis was no longer statistically significantly higher in the intervention arm (*p* = 0.92).

**Conclusions:**

As in comparable studies, we could not conclusively demonstrate the effectiveness of an HH intervention in reducing HAIs among residents of nursing homes, despite the use of clearly defined outcome measures, a standardized reporting instrument, and directly observed HH in a multicenter cluster RCT.

*Trial registration* Netherlands Trial Register, trial NL6049 (NTR6188). Registered October 25, 2016, https://www.trialregister.nl/trial/6049.

**Supplementary Information:**

The online version contains supplementary material available at 10.1186/s13756-021-00946-3.

## Introduction

Healthcare associated infections (HAIs) are a major cause of morbidity and mortality in nursing homes. The European Center for Disease Prevention and Control (ECDC) estimated an incidence of 3.2 HAI per 1000 resident days in long term health care in 2013 [1, 2]. Infection prevention measures, including improving hand hygiene (HH) compliance, can decrease HAIs [3]. Poor HH compliance by health care workers may result in higher rates of infections through the transmission of microorganisms from a health care worker to a resident and vice versa, and between residents, through either direct contact or fomite transmission.

While increased HH compliance of health care workers has been shown to decrease HAIs in hospitals, nursing homes, and the community setting, study outcomes are inconsistent [3–6]. A systematic review by Hocine, et al. from 2015, included 56 studies in nursing homes, of which 8 studies were randomized controlled trials (RCTs) [6]. Thirty-five studies (63%) reported results in favor of the HH intervention regarding infections of residents and/or staff. Of the 8 RCTs, only 2 concluded that increased HH was associated with a reduction of infections. The large variety in infections measured and methodological flaws limited the comparison between studies and the interpretation of the results. The authors concluded that future interventional studies should enhance methodological rigor by using clearly defined outcome measures, standardized reporting, and a relevant HH observation tool.

We evaluated the results of a multimodal HH trial (HANDSOME) tailored for nursing homes [7]. This was a large cluster RCT in 33 nursing homes in the Netherlands**.** The goal of this intervention was to increase the HH compliance of health care workers. The intervention was successful in increasing HH compliance in the intervention arm compared to the control arm: compliance in the intervention arm increased from 12% to 36% and in the control arm from 13% to 21% (OR: 2.28; CI: 1.67–3.11).

In this paper, we present the secondary outcome of the HANDSOME trial: the incidence of selected HAIs in residents of the nursing homes.

## Methods

HANDSOME is a cluster RCT in 66 Dutch nursing home units, designed to evaluate the effect of a multimodal intervention to increase health care workers’ HH compliance. Nursing homes in the intervention arm received the intervention while nursing homes in the control arm received no intervention. The trial was conducted from October 2016 through October 2017. The intervention took place from January through April 2017.

The multimodal intervention included a combination of activities for changing hygiene policy and the individual behavior of nurses. Nursing home policy changes were achieved by auditing personal hygiene rules as well as available HH materials. Nursing staff was subject to an e-learning, 3 live lessons, posters, and a photo competition [7, 8]. HH compliance was measured through unobtrusive direct observation according to the WHO-defined HH moments and recorded in a novel app [7, 8]. The nurses were blinded by giving distinct names to the lessons (The New Way of Working) and the observations (HANDSOME), so that they appeared to be different projects. Furthermore, nurses were told that the observers were registering the frequency of health care activities (in general).

Thirty-three nursing homes each committed 2 nursing home units to the study. Randomization was done per nursing home so that both units within one nursing home were always randomized to the same study arm. All nursing homes provided intense psychogeriatric and/or somatic care to geriatric residents. Units were defined as one to three wards within a nursing home. NH wards were considered eligible as a unit if they had three or more nurses working during observation hours (8 am to 1:30 pm on weekdays), so that we could observe a minimum of three nurses during one observation session. If there were not enough nurses employed during those hours in one ward, multiple wards were combined and considered one unit for purpose of this study. A nurse was defined as someone trained in nursing skills with either a 3-year nursing degree (*verzorgende*) or 4-year nursing degree (*verpleegkundige*). Nursing assistants (*helpenden*) were excluded. Nursing homes were computer randomized after baseline hand hygiene measurements to either the intervention arm or the control arm. Differences between the study arms were investigated, such as the level of care, type of care, the number of nurses per bed, and the availability of HH materials in the residents’ rooms. Size of the nursing homes was the only statistically significant background variable after randomization: the intervention arm had more small and medium-sized nursing homes (<88 beds, 88–118 beds) while the control arm had more large nursing homes (>118 beds). The study protocol, background information, and other results of the trial can be found elsewhere [7–10].

The outcome measures of this paper are the incidence of gastroenteritis, influenza-like illness (ILI), assumed pneumonia, urinary tract infections (UTIs), and infections caused by methicillin-resistant *Staphylococcus aureus* (MRSA) in nursing home residents. We investigated these HAIs based on the four most prevalent HAIs reported in nursing homes in Europe (respiratory disease, urinary tract infections, skin infections and gastroenteritis) [1]. We did not investigate skin infections since the incidence is low. We included MRSA since the incidence of MRSA in nursing homes in the Netherlands is of growing concern.

Residents’ infections in each unit were recorded weekly. Each nursing home unit had a staff member (nurse, team leader, or geriatrician) who recorded the incidence (per week) of gastroenteritis, ILI, pneumonia, UTI, and MRSA, in a notebook using the McGeer criteria [11]. MRSA is not defined by the McGeer criteria. It is generally tested in nursing homes with nasopharyngeal and oropharyngeal swabs and is per definition laboratory confirmed. Every infection per resident was recorded once in the study; multiple unique infections per resident could be recorded. All infection data were registered anonymously. Weekly illness incidence reports were sent to the researcher via email or WhatsApp. When the illness incidence report was not sent, the dedicated staff member at the nursing home unit received weekly reminders by email and/or phone until all illness incidence reports were collected. Units commenced their illness incidence reporting the same week that HH was first observed. The first observation of HH occurred over a period of 4 weeks in October 2016.

We compared our data to data from SNIV, a national surveillance network (www.sniv.nl). The SNIV routinely collects data from 34 nursing homes, representing approximately 4060 residents. No nursing homes participated simultaneously in this study and the SNIV surveillance program. At our request, SNIV provided infection incidence data corresponding with the weeks of the HANDSOME trial. This study and the SNIV both (1) use the McGeer illness definitions and (2) represent a geographically diverse sample of nursing homes throughout the Netherlands.

All nursing home units were included in the analyses in an intention-to-treat model. To calculate resident days, all beds in a unit were included, regardless of occupancy, since beds were generally all occupied during the study period [12]. Units were included for a particular week if the HAI-incidence was recorded for that week. Illness per 1000 resident days was calculated as (total recorded incidence per arm* 1000) / (total number of recorded resident-weeks per arm*7).

Differences in illness incidence between the intervention and control arms were explored per period: Baseline (October–December 2016); during the intervention (Follow-up 1: January-April 2017); and post-intervention (Follow-up 2: May–October 2017). Infection incidence rates in the intervention arm were compared to the control arm in a Poisson multilevel analysis to account for the clustering of observations within a nursing home. This model corrected for baseline differences (baseline infection incidence and the size of the nursing home, the only statistically significantly different background variable after randomization) as well as study period (baseline, during the intervention, follow-up) [7]. Since exceptionally high HAI incidence rates per unit per week could unduly affect the analyses, we performed a sensitivity analysis by rerunning the analyses after removing the highest 1% incident rates per HAI per week. We also tested if there was variation of treatment effect over time. We did this by replacing the variable “period” with “months” and adding an interaction term to the model (month * study arm).

Data from the control and intervention arms were combined to calculate yearly infection incidence rates per 1000 resident days (range, mean, and interquartile range) for the individual nursing home units over the period November 2016–October 2017, in order to ease comparison of our data to other datasets in the future. Data were analyzed using IBM SPSS Statistics for Windows, version 26 (IBM Corp., Armonk, N.Y., USA), and R version 4.0.2.

## Results

Of the 66 nursing home units in the HANDSOME trial, 36 (976 beds, median 25 per unit) were in the intervention arm, and 30 (886 beds, median 28 per unit) in the control arm. During the baseline measurements, the intervention and control arm units sent in their illness incidence reports on average 81% and 73% of the twelve weeks, respectively (Additional file 1: Table S1). Eight units (12%) left the study during the follow-up for various reasons: six intervention units (four during Follow-up 1 and two during Follow-up 2) and two control units (both during Follow-up 2) [7]. There was on average 99–100% reporting per week for both arms during the follow-up, excluding the weeks after units discontinued the study.

**Fig. 1:**
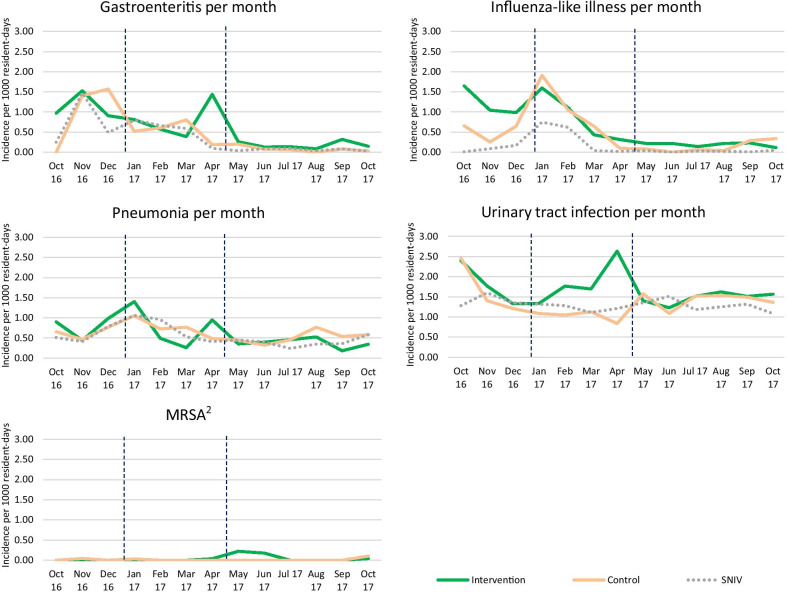
Infection incidence per 1000 resident days in nursing homes by month^1^ (n = 640,486 resident days). ^1^For comparison, incidence registered by the Dutch surveillance network for infectious diseases in nursing homes (SNIV) is also depicted (grey dotted line). The dashed vertical lines indicate the three study periods (Baseline, Follow-up 1, and Follow-up 2). ^2^SNIV did not provide data for MRSA

Figure 1 shows the incidence of episodes of gastroenteritis, ILI, pneumonia, UTI, and MRSA by study arm per month, covering 640,486 resident days. The infection incidence registered by SNIV is included for comparison. Our data showed similar trends to the SNIV data. Since MRSA was not a common cause of disease (16 cases of MRSA were reported, with one unit in the intervention arm reporting 8 cases in Follow-up 2), we excluded it from further analyses. The two arms in the study had similar rates of infection per month. There was evidence of variation of treatment effect over time for ILI (April *p*=0.01, May *p*=0.03, and August *p*=0.02), pneumonia (April *p*=0.01), and UTI (April *p*<0.001).

**Fig. 2:**
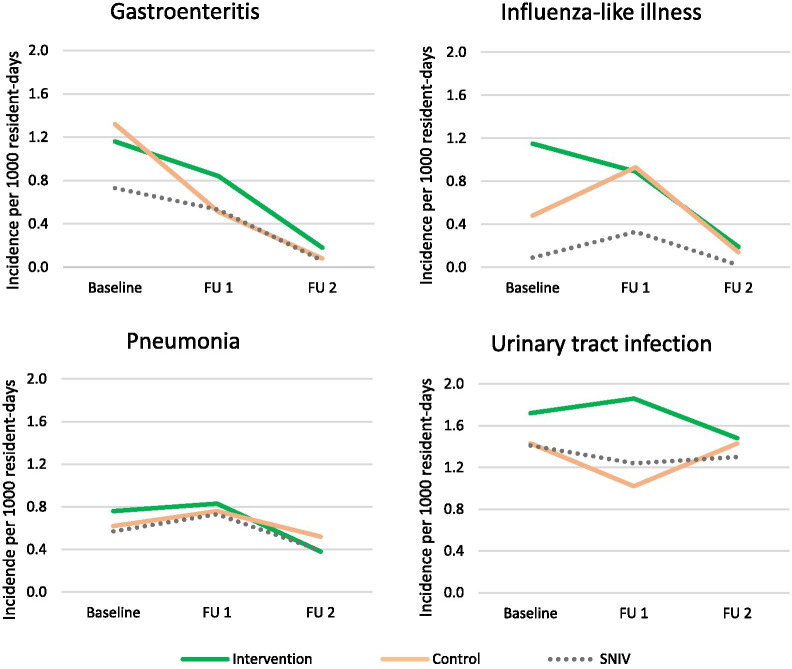
Infection incidence per 1000 resident days in nursing homes by study period^1^ (n = 640,486 resident days). Baseline: October 2016–December 2016, Follow-up 1: January 2017–April 2017, Follow-up 2: May 2017–October 2017. ^1^For comparison, incidence registered by the Dutch surveillance network for infectious diseases in nursing homes (SNIV) is also depicted (grey dotted line). FU: Follow-up

Figure 2 shows the same incidences, but now per study period, again including infection incidence registered by SNIV. There was more ILI in the intervention arm in the Baseline and more UTI in the intervention arm during Follow-up 1. In general, our study showed more reported infections than the SNIV, most notably for ILI.

Incidence fluctuated per intervention arm, HAI type, and period (Table 1). UTI was the most common infection reported (n=941), approximately triple the number of cases of pneumonia (n=392), ILI (n=346), or gastroenteritis (n=331). When analyzing the data in a multilevel Poisson analysis, the intervention arm had statistically significantly more gastroenteritis (*p*<0.001) and statistically significantly less ILI (*p*<0.01), when compared to the control arm. There were no statistically significant differences between the study arms with regards to pneumonia and UTI incidence. In the sensitivity analysis, there was no statistically significant difference between the study arms with regards to gastroenteritis (*p*=0.92).

**Table 1:** Healthcare-associated infection incidence per 1000 resident days in nursing homes: intervention versus control arm^1^

Illness and period^1^	Intervention arm		Control arm		Incidence rate ratio^2^ (95% CI) (Full dataset)		Incidence rate ratio (95% CI) Sensitivity analysis^2,3^	
	Per 1000 resident days	Number of cases	Per 1000 resident days	Number of cases		*p*-value		*p*-value
*Gastroenteritis*								
Baseline	1.16	(75/64617)	1.32	(72/54453)				
Follow-up 1	0.84	(89/106554)	0.51	(57/111048)				
Follow-up 2	0.18	(26/144914)	0.08	(12/158900)				
					2.32 (1.49, 3.61)	< 0.001	1.03 (0.56, 1.90)	0.92
*Influenza-like illness*								
Baseline	1.15	(74/64617)	0.48	(26/54453)				
Follow-up 1	0.89	(95/106554)	0.93	(103/111048)				
Follow-up 2	0.19	(27/144914)	0.14	(22/158900)				
					0.51 (0.31, 0.82)	< 0.01	–^4^	–
*Pneumonia*								
Baseline	0.76	(49/64617)	0.62	(34/54453)				
Follow-up 1	0.83	(88/106554)	0.76	(84/111048)				
Follow-up 2	0.38	(55/144914)	0.52	(82/158900)				
					0.87 (0.60, 1.26)	0.47	0.79 (0.52, 1.21)	0.28
*Urinary tract infection*								
Baseline	1.72	(111/64617)	1.43	(78/54453)				
Follow-up 1	1.86	(198/106554)	1.02	(113/111048)				
Follow-up 2	1.48	(214/144914)	1.43	(227/158900)				
					1.05 (0.78, 1.42)	0.75	1.15 (0.83, 1.59)	0.39

^1^Baseline: October 2016–December 2016, Follow-up 1: January 2017–April 2017, Follow-up 2: May 2017–October 2017. ^2^The results were corrected for the clustering of infection registrations within nursing homes, baseline differences and period in the study, in a multilevel Poisson regression. ^3^The sensitivity analysis excluded the highest 1% incident rates per HAI per week. ^4^Could not fit model due to convergence issues. CI: Confidence Interval

We explored how often nursing home units reported HAIs (i.e., reported a number other than zero). Other than UTI, each HAI type was reported in <10% of the weekly reports (Additional file 1: Table S2). UTI was reported in 23% of the weekly reports. Incidence of any HAI in any week was ≤16 per unit (79 per 1000 resident days).

There was a marked difference between the study arms in April 2017 (Follow-up 1) for gastroenteritis. This was largely caused by a gastroenteritis outbreak in one intervention unit in April 2017 (31 cases of gastroenteritis over 2 weeks (76 cases per 1000 resident days)), the most prominent outlier in the data. This is an outlier in our data since all other records of gastroenteritis per unit per month showed less than 17 cases per 1000 resident days. This increase in gastroenteritis coincided with a notable increase in UTIs in the same nursing home unit in the same 2-week period (27 cases of UTI per 1000 resident days).

Lastly, the yearly mean infection incidence rate (November 2016–October 2017) per 1000 resident days per unit was: 0.64 for gastroenteritis, 0.70 for ILI, 0.64 for pneumonia, and 1.63 for UTI (Table 2). By comparing the range and the interquartile range, we see that the data is skewed towards zero (Additional file 1: Table S2).

**Table 2:** Healthcare-associated infection rates per unit per 1000 resident days (November 2016–October 2017, n = 66 units)

HAI	Range	Mean	Interquartile range		
			25%	50% (median)	75%
Gastroenteritis	0–5.56	0.64	0.00	0.25	0.78
Influenza-like illness	0–5.72	0.70	0.00	0.30	0.92
Pneumonia	0–1.65	0.64	0.34	0.43	0.86
Urinary tract infection	0–6.28	1.63	0.72	1.26	2.18

## Discussion

We investigated the impact of an HH intervention for nursing homes staff on HAI in residents. Five illnesses were investigated: gastroenteritis, ILI, pneumonia, UTI, and MRSA. There was statistically significantly more gastroenteritis (*p*<0.001) and less ILI (*p*<0.01) in the intervention arm when compared to the control arm when taking baseline data into account and controlling for the clustering of observations in nursing homes, baseline differences, and the period in the study. For pneumonia and UTI, there were no differences between study arms. Sensitivity analysis did not confirm that there was statistically significantly more gastroenteritis in the intervention arm (*p*=0.92).

Other studies have also looked at the effect of an HH intervention on HAI reduction. The results of the systematic review cited in the introduction suggest that HH interventions may help control the infectious risk in nursing home settings, but that the precise impact remains poorly documented [6]. Many studies in the review were limited by methodological flaws; only 8 of 56 studies were RCTs, 6 of which were published over the last 5 years before the review (in the period 2010–2015). Also, most studies were in single-site nursing homes and provided a limited array of data. Finally, a low proportion of the studies in the review included direct observations of HH compliance, and the authors recommend strongly that future studies should include direct observation of HH compliance. Our current study complies with the recommendations from this review in that it is a large multicenter trial with extensive data collection on many possible determinants for HH compliance and risk factors for infection. Additionally, HH compliance was established through direct observation. Nevertheless, our study produced rather paradoxical results of which the interpretation is challenging.

After baseline, nursing homes were randomly assigned to either the control or intervention arm, ensuring that nursing homes from the same organization were in different study arms. Despite randomization, there were marked differences in the distribution of HAIs at baseline. This could possibly be explained by the fact that the introduction of infectious disease is a highly random phenomenon, especially when observed over a short period. Baseline differences between the two arms of the trial were particularly notable for ILIs and to lesser extent for gastroenteritis.

Many infectious diseases are seasonal. We addressed this through our RCT-design, assuming the seasonal changes to be the same in both arms. Yet, our implementation of the RCT may not have been ideal for two reasons: (1) It was possibly difficult to see a statistically significant difference between the study arms, because the follow-up period was primarily after the winter season when one would expect lower rates of gastroenteritis and ILI (February–October 2017); and (2) because of the generally low HAI incidence, observation is ideally performed over multiple years.

A hand hygiene intervention is not always the most important hygiene intervention to reduce HAIs, which can have both endogenous and exogenous sources. Hand hygiene compliance should primarily decrease HAIs that spread through person-to-person contact, with a secondary effect of lower contamination of surfaces. When the most prevalent transmission route is via droplet or aerosols, mask usage can be the most important hygiene intervention. We would therefore expect the effect of increased hand hygiene on gastroenteritis to be high and on pneumonia or UTI to be low, considering the disease pathways. At the same time, hand hygiene is necessary when handling a catheter and approximately 12% of nursing home residents have a catheter [13]. The results of our study are rather paradoxical (there was a statistically significant increase of gastroenteritis in the intervention arm) and emphasize that it is difficult to establish the effect of improved hand hygiene when using HAI as an outcome indicator.

To place the outcomes of the HANDSOME study into perspective, we compared these with a Dutch national surveillance program (SNIV) and European data from the ECDC. The nursing homes in HANDSOME (both intervention and control arms of the study) followed the SNIV data closely (except for ILIs); the control arm followed the SNIV trends more closely. A possible explanation that the nursing homes in the intervention arm registered more infections could be that the nursing homes in the intervention arm were extra alert to infections among residents because of the intervention and thus more motivated to provide diligent illness incident reports than nursing homes in the control arm. Comparing our data to the infection rates provided by the ECDC, we had slightly more reporting of HAI (4.2 per 1000 resident days vs. 3.2 per 1000 resident days), even though the ECDC has a broader definition of HAI, including, for instance, skin/soft tissue infections, eye/ear/mouth/nose infections and bloodstream infections [1].

We used the McGeer criteria in this study to define infectious diseases for two reasons: (1) the national SNIV uses the McGeer criteria, and we wanted to compare our data to another dataset; and (2) it is hard to justify (invasive) diagnostic testing in nursing home residents when the goal of the study is not to find suspected HAIs but to understand the effect of hand hygiene on HAIs. At the same time, the diagnosis of HAIs is often uncertain and may be based on subjective criteria. Additionally, the McGeer criteria have been updated by diverse researchers and organizations; newer insights could lead towards more accurate identification of HAIs [14, 15]. Future studies could perform diagnostics for more definitive results or use updated versions of the McGeer criteria.

The effect of HH on HAIs may be dependent on various infection prevention measures, such as cleaning methods and schedules. It is also assumedly dependent on the HH compliance level. Although the HANDSOME intervention was successful in tripling the HH compliance in the intervention arm, it only reached a 36% compliance rate [7]. The hand hygiene compliance in the intervention arm may not have crossed a critical threshold to lower infection rates. Some (primarily single-site or small-scale) studies in nursing homes have shown a correlation between HH compliance and infection rates, although larger studies generally show no relationship, making it difficult to determine a threshold value [6]. The compliance rate after the intervention might have been higher if more nurses had attended the lessons; the estimated attendance of health care workers at *at least one* of the lessons varied per unit: 23% units had <50% of the unit’s health care workers attending at least one lesson, 18% had 50–74% attendance at *at least one* lesson and 59% had >75% attendance at *at least one* lesson (n=22).

Understanding the pathways of HAIs during social interactions in nursing homes is also important when evaluating the results of interventions on HAIs. In contrast to hospital settings, nursing homes promote the socialization of residents. Residents may practice poor hygiene, and hence infect each other. The HANDSOME intervention did not target residents. Therefore, it cannot be expected that the direct resident-to-resident infection rate decreased. There are also social interactions in a nursing home between residents and staff for which HH is not prescribed by the WHO, such as a handshake or patting a hand [16]. This is different than in a hospital, where all hand interactions are considered HH opportunities [17].

A strength of the study is that it is based on data from a large multicenter cluster RCT. There are also limitations. Firstly, there may have been factors that influenced the reliability of the HAI data. Illness was recorded weekly by hand, which could elicit recall bias. Although the nursing home staff was accustomed to reporting infections in individual dossiers, they were not accustomed to reporting weekly infections for the unit as a whole. Since this type of illness incident reporting was new, it may have taken time until the illness incident reporting was consistent. Secondly, consistency between units may also have been a problem, since the function of the staff member who registered illnesses (nurse, team manager, or geriatrician) varied per unit. The staff member’s knowledge of HAIs present in the unit may also have differed. At the same time, this study used stratified randomization; for every nursing home in the intervention arm (2 units), there was generally one nursing home from the same organization in the control arm (2 units), thereby minimizing differences between study arms. The nursing homes in the two study arms were also not statistically significantly different for various variables, including management style, number of nurses per resident, and the intensity of care [7]. Therefore, we expect the illness incident reporting errors to be similar in the two arms of the study.

## Conclusion

This study, similarly to comparable studies, could not conclusively demonstrate the effectiveness of an HH intervention in reducing HAIs among residents of nursing homes, despite the use of clearly defined outcome measures, a standardized illness incident reporting instrument, and directly observed HH in a multicenter cluster RCT. This could be due to an insufficient increase in HH compliance and/or other factors in the nursing home environment that need to be addressed concurrently in order to decrease illness rates.

## Supplementary Information

**Additional file 1.** Weekly incidence detail reports.** Table S1**. Number of received illness incident reports from nursing home units per study week.** Table S2**. Number of cases (incidence) of a HAI per unit per week (intervention n=1612 weeks, control n = 1477 weeks).

## Data Availability

The datasets used and/or analyzed during the current study are available from the Erasmus MC, department of public health on reasonable request.

## Abbreviations

CI: Confidence interval
FU: Follow-up
HAI: Healthcare-associated infection
HH: Hand hygiene
ILI: Influenza-like illness
OR: Odds ratio
RCT: Randomized controlled trial
UTI: Urinary tract infection
