# Supplementary material for: The effect of a hand hygiene intervention on infections in residents of nursing homes: a cluster randomized controlled trial
Source: Antimicrob Resist Infect Control. 2021 May 20;10:80. doi: 10.1186/s13756-021-00946-3 (PMC8138990; doi:10.1186/s13756-021-00946-3)
Supplement: Supplementary file 1 — Additional file 1. Weekly incidence detail reports. Table S1. Number of received illness incident reports from nursing home units per study week. Table S2. Number of cases (incidence) of a HAI per unit per week (intervention n=1612 weeks, control n = 1477 weeks). [file 13756_2021_946_MOESM1_ESM.pdf]

Additional file: Weekly incidence detail reports

Teesing *et al.* The effect of a hand hygiene intervention on infections in residents of nursing homes: a cluster randomized controlled trial. *Antimicrob Resist Infect Control* doi:10.1186/s13756-021-00946-3

Table S1: Number of received illness incident reports from nursing home units per study week

Week, Year	Intervention arm	Control arm
41, 2016	3	0
42, 2016	15	1
43, 2016	26	8
44, 2016	33	19
45, 2016	34	27
46, 2016	33	29
47, 2016	33	29
48, 2016	33	30
49, 2016	35	30
50, 2016	35	30
51, 2016	35	30
52, 2016	35	30
1, 2017	34	29
2, 2017	34	29
3, 2017	34	30
4, 2017	34	30
5, 2017	34	30
6, 2017	33	30
7, 2017	34	30
8, 2017	34	30
9, 2017	34	30
10, 2017	33	30
11, 2017	32	30
12, 2017	32	30
13, 2017	32	30
14, 2017	32	30
15, 2017	32	30
16, 2017	32	30

Week, Year	Intervention arm	Control arm
17, 2017	32	30
18, 2017	32	30
19, 2017	32	30
20, 2017	32	30
21, 2017	32	30
22, 2017	32	30
23, 2017	32	30
24, 2017	32	30
25, 2017	32	30
26, 2017	32	30
27, 2017	32	30
28, 2017	32	30
29, 2017	32	30
30, 2017	32	30
31, 2017	32	30
32, 2017	32	30
33, 2017	32	30
34, 2017	32	30
35, 2017	32	30
36, 2017	32	30
37, 2017	32	30
38, 2017	30	28
39, 2017	30	28
40, 2017	30	28
41, 2017	30	28
42, 2017	30	28
43, 2017	30	28
44, 2017	30	28

Table S2: Number of cases (incidence) of a HAI per unit per week (intervention n=1612 weeks, control n=1477 weeks)

Number of incidences per week per unit	Gastroenteritis		Influenza-like illness		Pneumonia		Urinary tract infection		MRSA	
	Int.	Control	Int.	Control	Int.	Control	Int.	Control	Int.	Control
0	1494	1413	1495	1395	1458	1323	1227	1151	1607	1472
1	93	35	82	52	139	130	303	269	3	5
2	12	13	18	17	7	21	63	46	0	0
3	8	8	11	6	6	3	14	9	0	0
4	2	2	2	3	0	0	4	1	2	0
5	0	1	2	2	1	0	0	0	0	0
6	0	2	1	1	1	0	0	0	0	0
7	1	0	1	0	0	0	0	1	0	0
8	0	1	0	1	0	0	0	0	0	0
9	0	0	0	0	0	0	0	0	0	0
10	0	1	0	0	0	0	0	0	0	0
11	0	1	0	0	0	0	1	0	0	0
12	0	0	0	0	0	0	0	0	0	0
13	0	0	0	0	0	0	0	0	0	0
14	0	0	0	0	0	0	0	0	0	0
15	1	0	0	0	0	0	0	0	0	0
16	1	0	0	0	0	0	0	0	0	0
Percentage of weeks with non-zero HAI incidence	93%	96%	93%	94%	90%	90%	76%	78%	100%	100%
Range of incidence per week per 1000 resident days	0-79	0-52	0-57	0-38	0-24	0-18	0-54	0-71	0-16	0-10

Int.: Intervention
